# Visual Responses of Photoreceptor-Degenerated Rats Expressing Two Different Types of Channelrhodopsin Genes

**DOI:** 10.1038/srep41210

**Published:** 2017-01-23

**Authors:** Masatoshi Sato, Eriko Sugano, Kitako Tabata, Kei Sannohe, Yoshito Watanabe, Taku Ozaki, Makoto Tamai, Hiroshi Tomita

**Affiliations:** 1 Laboratory of Visual Neuroscience, Graduate Course in Biological Sciences, Iwate University Division of Science and Engineering, 4-3-5 Ueda, Morioka, Iwate 020-8551, Japan; 2 Soft-Path Engineering Research Center (SPERC), Iwate University Division of Science and Engineering, Morioka 020-8551, Japan; 3 Tohoku University Graduate School of Medicine, 1-1 Seiryo, Aoba, Sendai, Miyagi 980-8574, Japan; 4 Clinical Research, Innovation and Education Center, Tohoku University Hospital, 1-1 Seiryo, Aoba, Sendai, Miyagi 980-8574, Japan

## Abstract

Optogenetic technologies are expected to be applicable for clinical use in restoring vision. However, the degree of recovered visual function is highly dependent on the function of the chosen optogenetic gene. To investigate the effect on visual function of dual expression of genes with different wavelength sensitivities, we transduced a modified *Volvox*-derived channelrhodopsin gene (*mVChR1*) via an adeno-associated virus vector into transgenic rats harbouring the *ChR2* gene in retinal ganglion cells. These transgenic rats were given an intraperitoneal injection of N-methyl-N-nitrosourea to induce the degeneration of native photoreceptor cells prior to transduction of *mVChR1*. Optical coherence tomography images indicated the degeneration of the native photoreceptor cells after the N-methyl-N-nitrosourea injection. Complete loss of function of the native photoreceptor cells was confirmed using electroretinograms. In the *ChR2* transgenic rats, visually evoked potentials were clearly detectable in spite of native photoreceptor function abolishment; however the responses were limited to within blue wavelengths. In contrast, the limited wavelength sensitivities were improved by the additional transduction of *mVChR1*, which exhibited sensitivities to green and red. Thus, the transductions of dual genes encoding channelrhodopsins that exhibit different wavelength sensitivities represents a promising candidate method to expand and to enhance rescued wavelength sensitivities in blind subjects.

Channelrhodopsin-2 (ChR2), which functions as a light activated cation-selective ion channel, plays a central role in optogenetic technologies^1^,^2^,^3^. Since the discovery of ChR2, various additional types of optogenetic genes including those encoding light-gated chloride channels^4^ have also been identified, which have allowed the subsequent exploration of new strategies such as the optical control of neural^2^ and behavioural activity^5^, and studies of brain function^6^, mechanism of neuronal diseases^7^, and vision restoration^8^ across various fields of life sciences.

We previously reported that visual^9^ and behavioural^10^ responses in blind rats could be restored by gene transfer of ChR2. Safety studies were also performed in rats and marmosets^11^, which showed that no immunological responses^12^ were induced by the continuous expression of a *ChR2* gene that was derived from the unicellular green algae *Chlamydomonas reinhardtii*^13^,^14^, rather than from a human source. Gene therapy using *ChR2* thus represents a potential method for restoring vision in blind patients^15^. However, ChR2-expressing rats exhibiting photoreceptor degeneration could only detect wavelengths less than 540 nm because of the limitation of the ChR2 action spectrum^16^. Thus, recovered visual function depends on the protein function of the gene utilised for gene therapy. Alternatively, one possible method to resolve the limited wavelength sensitivity would be to incorporate another channelrhodopsin (ChR) such as *Volvox*-derived ChR1 (VChR1)^17^. Toward this end, we generated a modified *Volvox* channelrhodopsin-1 (mVChR1) protein that exhibited a broad spectrum and showed that the blind rats transduced with the *mVChR1* gene could record visually evoked potentials with stimulation at 450–600 nm and that optomoter responses were elicited with the all of colour stripes, i.e., blue, green, and red^18^.

Therefore, to potentially enhance the restored light sensitivities in the subject animals, we investigated the effects of dual *ChR2* and *mVChR1* gene expression on the visual function of blind rats. In the current study, we initially planned to transduce both genes by using an AAV vector. However the application of the mixed AAV vectors carrying *ChR2* and *mVChR1* exhibited low transduction efficiency of each gene. Consequently, we utilised our previously developed transgenic rats harbouring the *ChR2* gene regulated by the thy-1.2 promoter (TG-ChR2), and abolished the function of the native photoreceptor cells via intravenous injection of N-methyl-N-nitrosourea (MNU). Notably, the light sensitivities of photoreceptor-degenerated TG-ChR2 rats were improved over the wavelengths wherein the ChR2 exhibits less sensitivity by the additional transduction of *mVChR1*. Thus, the transduction of more than one gene with varying properties may yield additive effects in the process of gene therapy for restoring vision.

## Results

### Photoreceptor degeneration in TG-ChR2 rats

**Figure 1:**
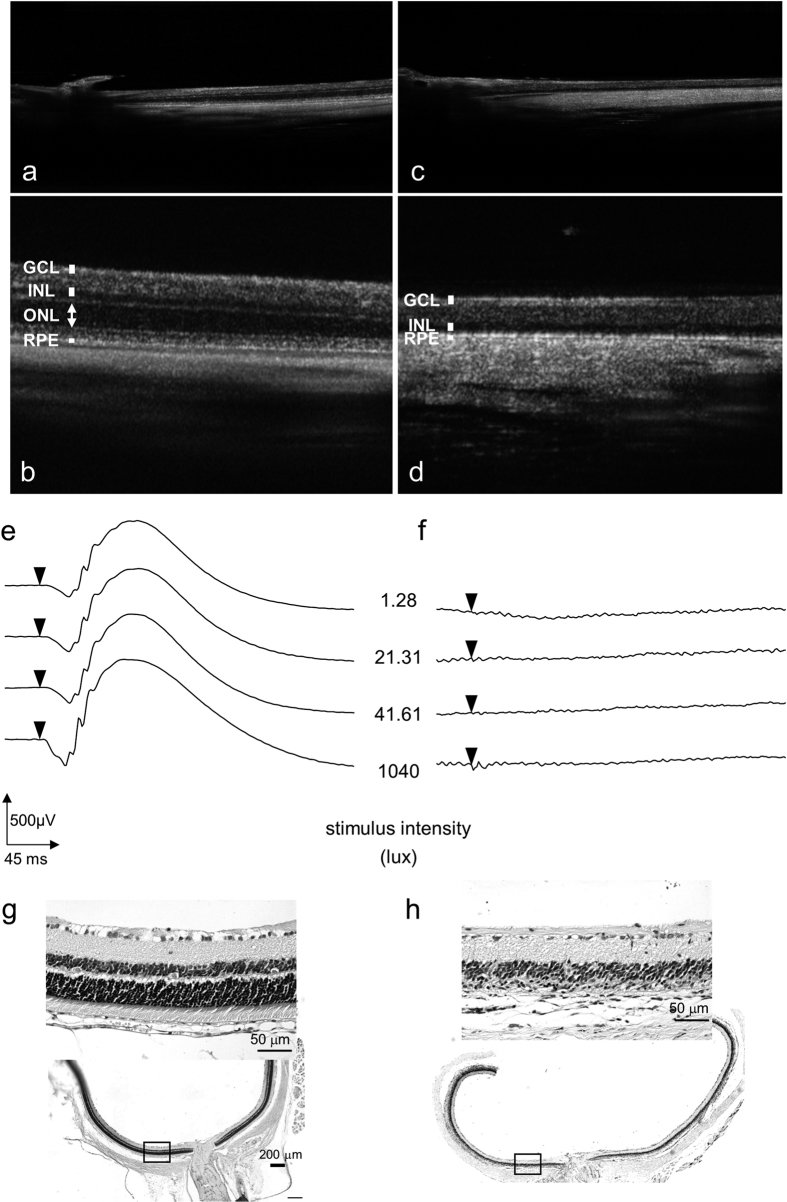
Photoreceptor degeneration induced by an intraperitoneal injection of MNU. OCT images were scanned from a wild type rat (**a**,**b**) and a rat 7 days after MNU injection (**c**,**d**). The wide field images including the optic nerve were shown in (**a**,**c**). The outer nuclear layer (ONL; arrow) was present in the retina of a normal rat, whereas the ONL had not observed in the retina of the rat 7 days after MNU injection. The vertical bar indicate the thickness of each layer. ERGs evoked by a white LED flash at various intensities from a normal rat (**e**) and a rat 7 days after MNU injection (**f**) are shown. The paraffin-embedded retinal sections from a normal (**g**) and an MNU-treated (**h**) rat were stained with haematoxylin and eosin.

Optical coherence tomography (OCT) images were scanned on the retina from a wild type rat and from a rat 7 days after MNU injection. The outer nuclear layer (ONL) was well-organised in the retinas of the wild type rat (Fig. 1a,b) and TG-ChR2 rat (data not shown), whereas the ONL had almost disappeared across an extensive area in the retina of the MNU-injected rat (Fig. 1d). The electroretinograms (ERGs) from the normal rat showed a clear light intensity response (Fig. 1e); however, light response from the MNU-treated retina was undetectable even following stimulation at the maximum intensity (Fig. 1f). Together, these morphological and functional findings were taken as confirming photoreceptor degeneration in the MNU-treated rats. Histological examination also demonstrated that the photoreceptor degeneration had occurred throughout extensively throughout the retina (Fig. 1h).

### Recording of visually evoked potentials (VEPs)

**Figure 2:**
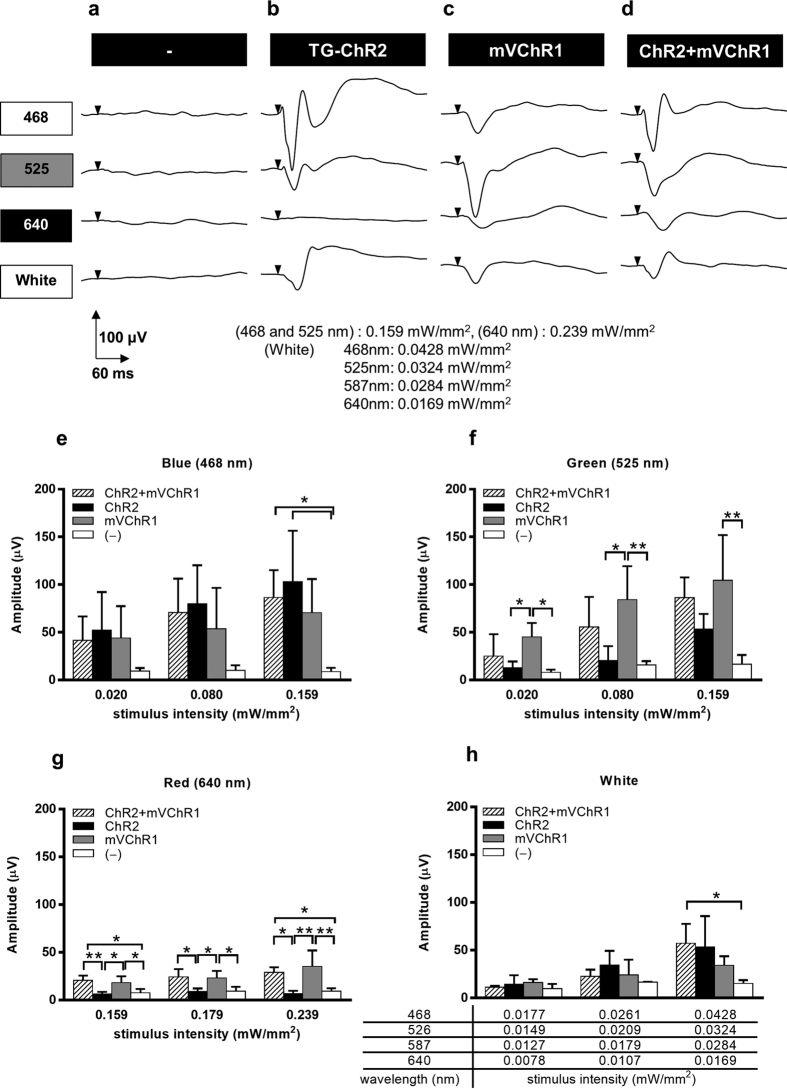
Recordings of VEPs with stimuli from blue-, green-, red-, and white-LEDs. Photoreceptor-degenerated wild-type rats (−) did not respond to any coloured LEDs (**a**). Photoreceptor-degenerated TG-ChR2 rats clearly responded to stimulus from blue-LEDs (**b**). Photoreceptor-degenerated-*mVChR1*-transduced wild-type (**c**) and TG-ChR2 rats (**d**) responded to all coloured LEDs. Evoked potentials in response to blue (**e**), green (**f**), red (**g**), or white LED light (**h**) are shown. Data are represented as the means ± SD (*P < 0.05, **P < 0.01). n = 3 in *mVChR1* and (−) with white LED, n = 4 in the others.

**Figure 3:**
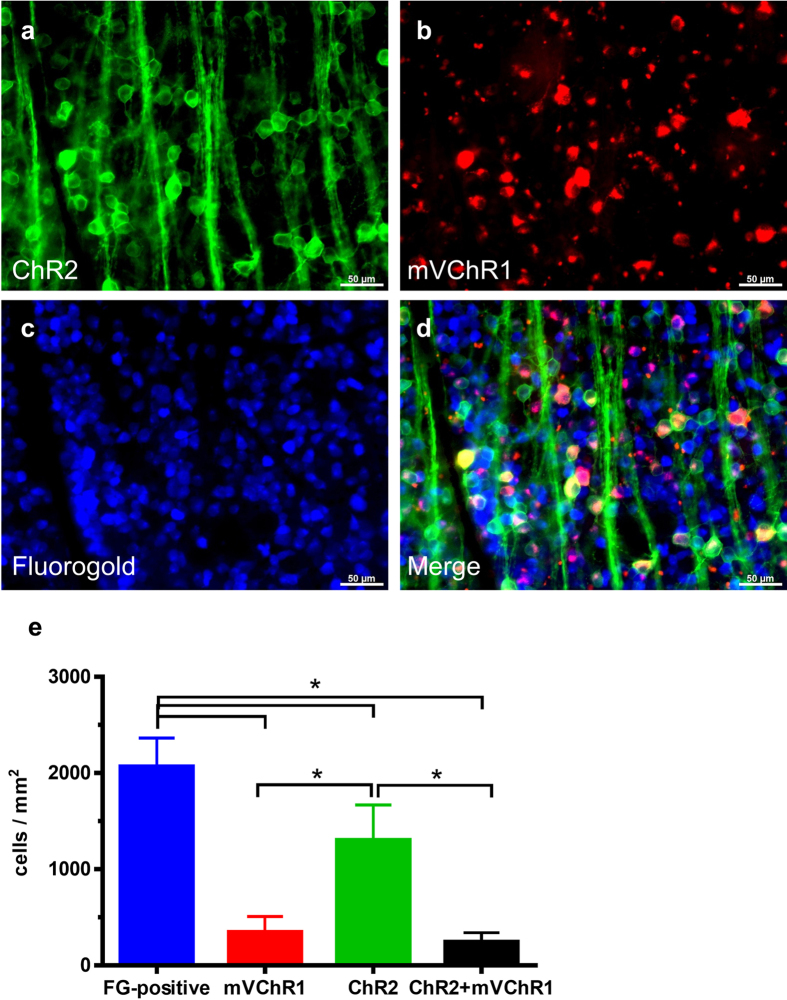
Number of *ChR2*- and *mVChR1*-expressing cells in rat retinas. The green (**a**) and red (**b**) fluorescence corresponds to *ChR2* and *mVChR1* expression, respectively. The RGCs (**c**) were retrograde-labelled with fluorogold (scale bar = 20 μm). The merged image is shown in (**d**). Data are represented as the means ± SD (**P < 0.01, n = 4).

VEPs using various coloured light emitting diodes (LEDs) elicited no responses from wild- type rats following MNU injection (Fig. 2a); however, VEPs from the photoreceptor-degenerated TG-ChR2 rats were still recorded upon exposure to the various light stimuli except for the red LED (Fig. 2b). Notably, VEPs from the photoreceptor-degenerated wild-type rats were recovered after the intravitreous injection of the AAV-mVChR1 vector (Fig. 2c). Consistent with this, a VEP waveform following red LED stimulus was clearly observed following the intravitreous injection of AAV-mVChR1 into the photoreceptor-degenerated TG-ChR2 rats (Fig. 2d), although they exhibited no response to red LED stimulus prior to the injection (Fig. 2b). No significant differences in the VEPs to blue or white LEDs were noted (Fig. 2e,h). However, the VEPs stimulated using a green or red LED showed some characteristic features (Fig. 2f,g). Specifically, the sensitivity of the ChR2 protein to green wavelengths was weaker than that of mVChR1 but the response was reinforced by the additional expression of mVChR1 in the TG-ChR2 rat (Fig. 2f). This phenomenon was also observed in the sensitivity to red wavelengths (Fig. 3g) but not to blue wavelengths (Fig. 2e).

### Expression profiles of ChR2 and mVChR1 in the retina

In the TG-ChR2 rats, venus fluorescence indicative of *ChR2* expression (originating from a venus/ChR2 gene fusion) was extensively observed in the flat-mounted retina, as previously reported^10^. The ganglion cells in the TG-ChR2 rats intravitreously injected with the AAV-mVChR1 were found to express the *ChR2* and *mVChR1* genes, as expected. To facilitate investigation of the number of RGCs expressing each gene, these genes had been fused to the venus and pmCherry florescence reporter genes, respectively. In addition, the RGCs were retrograde-labelled with a fluorescent tracer, Fluorogold (Fig. 3a). The number of RGCs, mVChR1-expressing RGCs, ChR2-expressing RGCs, and RGCs expressing both ChR2 and mVChR1 were 2068 ± 296, 346 ± 162, 1304 ± 365, and 247 ± 95 cells/mm^2^ (Fig. 3b), respectively. The ratios of mVChR1-, ChR2- s, and both ChR2 and mVChR1-expressing RGCs among all RGCs were 16.7 ± 4.8%, 63.1 ± 7.5%, and 11.9 ± 2.5%, respectively.

### Patch clamp recordings

**Figure 4:**
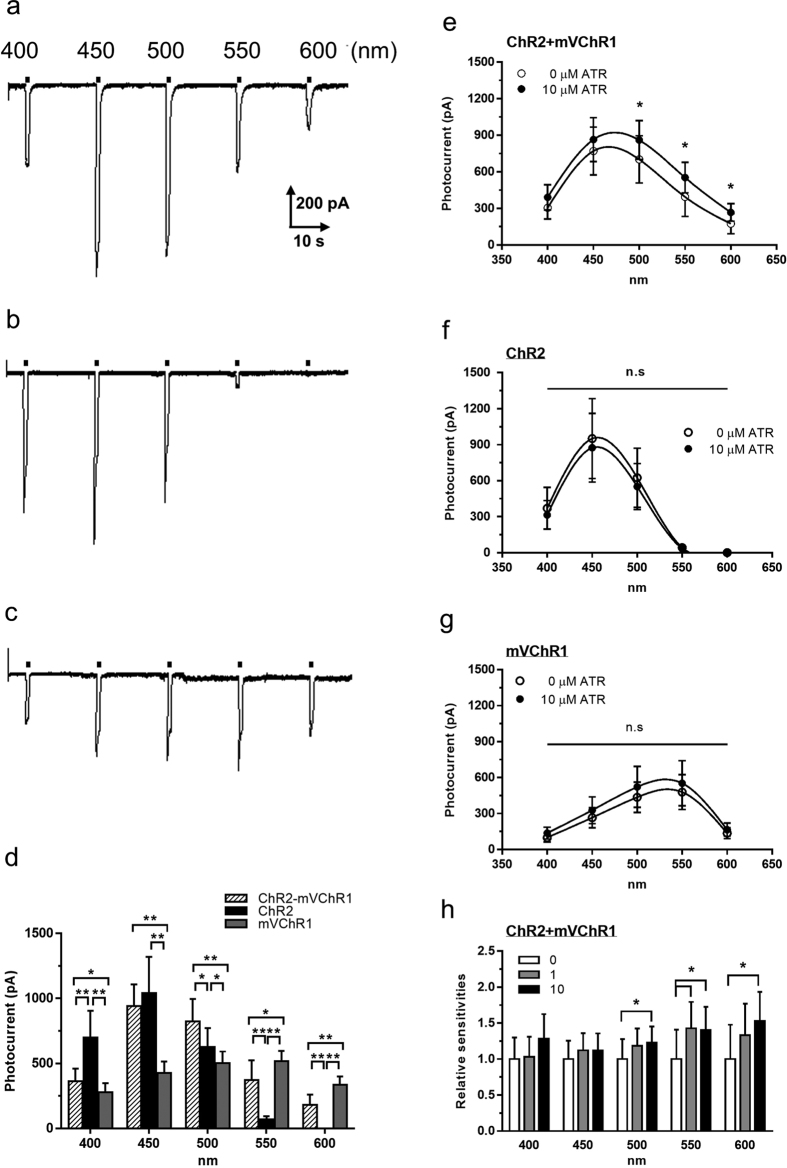
Photocurrent in the cells expressed both ChR2 and mVChR1 gene. Typical waveform of the cells expressing both *ChR2* and *mVChR1* (**a**), ChR2-expressing cells (**b**), and *mVChR1*-expressing cells (**c**). Wavelength sensitivities are shown in (**d**). Effects of ATR on the photocurrents in the co-expressing (**e**), ChR2-expressing (**f**), and mVChR1-expressing (**g**) cells following various wavelength stimuli. Increased photocurrents were observed at stimuli over 500 nm in a dose dependent manner (**h**). Data represent the means ± SD (n = 9).

To investigate the effects of expression of both genes on the photocurrent, we established cell lines that stably expressed either or both of the *ChR2* and *mVChR1* genes. Cells expressing both *ChR2* and *mVChR1* responded to 400–600 nm of light (Fig. 4a), with the peak wavelength of ChR2 being approximately 450 nm although it did not demonstrate sensitivity over 550 nm (Fig. 4b) as was observed for mVChR1 (Fig. 4c). Increased photocurrent mediated by dual gene expression was observed in wavelength ranges such as 550 and 600 nm, in which ChR2 did not exhibit effective functioning. However, the photocurrents in the 550 and 600 nm range in human embryonic kidney (HEK) − ChR2 + mVChR1 cells were significantly lower than those in HEK-mVChR1 cells (Fig. 4d). Therefore, we investigated the effect of the all-trans form of retinal (ATR), a chromophore for the ChRs, on the photocurrents. Following treatment, the photocurrents in the cells expressing both genes significantly increased at the wavelengths reflecting mVChR1 sensitivities (Fig. 4e) in an ATR dose-dependent manner (Fig. 4h). Conversely, the addition of ATR exhibited no effect on the photocurrents in the ChR2- (Fig. 4f) and mVChR1-(Fig. 4g) expressing cells.

## Discussion

Retinitis pigmentosa (RP) is one of several diseases that cause blindness by a gradual death of cone cells following the death of rod photoreceptors. Various mutations of genes mostly related to retinal phototransduction pathways have been identified in these disorders; however, effective treatments have not yet been developed. Notably, however, within the eyes of patients with RP, the inner retinal neurons such as RGCs and bipolar cells remain well-preserved after causing the death of photoreceptor cells^19^,^20^,^21^. Various animal models of photoreceptor degeneration have been established, such as rats and mice that carry the same mutation as human RP representing spontaneous^22^ and transgenic^23^,^24^,^25^ models, or result from light^26^,^27^- or chemical^28^,^29^-induced retinal degeneration. In the current study, the MNU-induced model was chosen because the transgenic rat model required substantial time for degeneration to occur and the superior and inferior parts of the retina exhibit differing susceptibilities to light-induced degeneration^30^. In contrast, intraperitoneal injection of MNU leads to extensive photoreceptor degeneration^31^ that is not dependent on the rat strain^32^. Here, we demonstrated that ERGs were completely abolished by MNU-injection (Fig. 1f,f); however, TG-ChR2 rats still exhibited VEPs because ChR2 was expressed in the RGCs (Fig. 2b).

Gene therapy that targets *ChR* gene transduction into surviving neurons, RGCs, or bipolar cells is expected to comprise a promising therapy for restoring vision to blind patients, wherein the restored visual function would likely be dependent on the function of the encoded protein. Our developed mVChR1 channel exhibits broader wavelength sensitivities than ChR2. However, the light sensitivity was less than that observed in the normally functioning retina. The additional gene transduction of *mVChR1* onto photoreceptor-degenerated TG-ChR2 retinas did not enhance the light sensitivity within the sensitive peak wavelengths of ChR2 (Fig. 2e). Similarly, although ChR2 demonstrated a weak sensitivity to wavelengths around 525 nm, the additive expression of ChR2 and mVChR1 did not increase the light sensitivity at the sensitive peak wavelength of mVChR1 (Fig. 2f). One reasonable possibility for the lack of observed additive effect following dual gene expression may be that each gene was expressed in different cells. However, the following analysis demonstrated that this was unlikely to be the case. As shown in Fig. 3, *ChR2* expression was observed in approximately 63% of total RGCs, which is higher than that observed in our previous study^10^. In the current study, we counted the cells not only on the surface of the flat-mounted retina but also within the depths by using a Z-axis scanning method, which allowed us to accurately determine the number of positive cells. Approximately 16.7% of mVChR1-positive cells driven by the CAG promoter and approximately 63.1% of ChR2-positive cells driven by the ganglion cell specific promoter, thy-1.2 were observed in the retina. The estimated number of co-expressing RGCs from the each percentage is 10.5% (0.167 × 0.631) that almost similar rate with the counting number of co-expressing cells (11.9%). These results indicated that the mVChR1 gene expression driven by the CAG promoter by the intravitoreous injection of AAV was specifically induced in the RGCs.

To further investigate the potential mechanism underlying the lack of additive effects at sensitive wavelengths, we established cell lines stably coexpressing ChR2 and mVChR1 and investigated the photocurrent using LEDs at various wavelengths. We observed similar responses as found in the *in vivo* studies (Fig. 4). The photocurrents in the cells expressing both genes were significantly lower upon 550 and 600 nm stimuli than those in HEK-mVChR1 cells. We therefore considered the possibility that the concentration of the photoisomerisable chromophore ATR, well-known to be required for ChR function^33^,^34^ and naturally existing in mammalian tissue^1^,^35^, might not be sufficient to adequately support full ChR activity in the coexpressing cells. Consistent with this supposition, we found that the addition of ATR was effective in increasing the photocurrents around the mVChR1-sensitive wavelengths although the photocurrents in HEK-ChR2 and -mVChR1 single ChR-expressing cells were not affected (Fig. 4g,h), indicating that the endogenous ATR was insufficient in co-expressing cells, especially for mVChR1. In addition, ATR might also exhibit some differences in affinity toward ChR2 and mVChR1.

In summary, we demonstrated that the transduction of an additional gene was able to improve the wavelength sensitivities in transduced retinas through incorporating different ChR properties. Currently, gene therapy using ChRs is on-going worldwide and multiple new optogenetic genes^36^,^37^ are being developed in the laboratory. However, there remains a gap between the findings of basic research and their clinical application. Our results that the recovered visual function effected by gene therapy would likely be highly dependent on the gene used to transduce the retina and the proposed strategy to maximise this effect provide important information for the design of gene therapy protocols to optimise patient outcome.

## Materials and Methods

### Animals

All experimental procedures were approved by the Institutional Animal Care and Use Committee (No. A201505), and Gene Recombination Experiment Safety Committee (No. 201508) of Iwate University. All animal experiments were conducted in accordance with the guidelines of the Animal Care and Use Committee of Iwate University, Japan. Rats were kept in cyclic light *ad libitum* with free access to water. For this study, we utilised the thy1.2-ChR2V transgenic (TG-ChR2) rat line 4, which was previously established in our laboratory and has been shown to constitutively express the *ChR2* gene in RGCs^10^. Heterozygous TG-ChR2 rats were crossed with Wistar (wild type) rats (CLEA Japan, Inc. Tokyo) and screened by genomic polymerase chain reaction (PCR) for the presence of the transgene. Briefly, tail biopsies from two week-old rats were performed and genomic DNA was extracted from the tail using the DNeasy Blood and Tissue kit (QUIAGEN, Tokyo, Japan) according to manufacturer instruction. The forward (5′-AGCTGATCTGCACCACCG-3′) and reverse (5′-TCCTTGAAGAAGATGGTGCG-3′) primers for the cDNA sequence of Venus protein were used for the PCR reaction with a Real-Time PCR-CFX ConnectTM Real-Time System (Bio-Rad, Tokyo, Japan). The melting curve of each PCR product was analysed using Precision Melt AnalysisTM software for the detection of rats carrying the *ChR2V* gene.

### Induction of photoreceptor degeneration

Rats were administered a single intraperitoneal injection of MNU (Sigma-Aldrich, St. Louis, MO) at a dose of 60 mg/kg body weight. The MNU solution was freshly prepared with sterile physiological saline containing 0.05% acetic acid immediately prior to use and stored at 4 °C in the dark.

### OCT imaging

Rats were anesthetised by intramuscular injection of ketamine (75 mg/kg) and medetomidine (0.5 mg/kg) and their pupils were dilated with tropicamide (Midrin-P, Santen Co., Ltd., Osaka, Japan). The eye was placed under local anaesthesia using oxybuprocaine (Santen Co., Ltd., Osaka, Japan) and covered with a contact lens. Image acquisition of 1.1 mm-length of the rat retina including the optic disk was performed using the line scan mode on an OCT imaging device equipped with a special ordered lens (RS-3000, NIDEK Co., Ltd., Aichi, Japan).

### Histology

At 7 days following the MNU-injection, eyes from some of the rats (2 each from the control and MNU-injected animals) were enucleated and immersed in 4% paraformaldehyde in phosphate buffered saline (PBS) overnight at 4 °C and then embedded in paraffin. Serial sections (4 μm) of whole eyes were cut sagittally through the cornea and parallel to the optic nerve, stained with haematoxylin and eosin, and then analysed by microscopy (BZ-9000; Keyence, Tokyo, Japan).

### Recording of ERGs and VEPs

ERGs and VEPs were recorded as described previously^12^,^15^,^16^,^38^. Briefly, rats were dark-adapted overnight. A small contact lens with an electrode was then mounted on the cornea and a reference electrode (a 27-gauge needle) was placed subcutaneously on the bridge of the nose. Photic stimuli were generated using a white LED. The photic stimuli were applied for 5-ms duration. The high- and low-path filters were set to 1 kHz and 0.5 Hz, respectively. Three consecutive response waveforms were averaged for each ERG measurement. For VEPs, recording electrodes (stainless screws) were embedded epidurally on each side 6.8 mm behind the bregma and 3 mm lateral of the midline, and a reference electrode (a stainless screw) was embedded epidurally on the midline 11.8 mm behind the bregma at least 7 days prior to the experiments. Under ketamine-medetomidine anaesthesia, the pupils of rats were dilated with 0.5% tropicamide and 0.5% phenylephrine hydrochloride. The eyes of the rats were subjected to photic stimuli of various intensities using different colour LEDs (Blue: 468 nm, Green: 525 nm, Red: 640 nm), applied for 10-ms duration with a frequency of 1 Hz. The high- and low-path filters were set to 50 Hz and 0.05 Hz, respectively. Each VEP response was recorded 200 times, consecutively, and then response waveforms were averaged for each VEP measurement.

### Preparation of the adeno-associated virus vector

Plasmid vectors containing *mVChR1* were used for production of the adeno-associated virus vector. To visualise the gene expression, venus or pmCherry cDNA was fused to *mVChR1* cDNA. The basic construction of the plasmid vector containing a hybrid CMV enhancer/chicken β-actin promoter (CAG) has been previously described^8^,^9^. The AAV Helper-Free System (Agilent Technologies, Santa Clara, CA) was used to produce infectious AAV virions according to manufacturer instruction. AAV vectors were purified using a slight modification of a previously described single-step column purification method^39^. The concentration of the purified AAV vectors was determined by measuring AAV capsid protein levels using ELISA (PROGEN, Heidelberg, Germany).

### Intravitreal injection of adeno-associated virus

Rats were anaesthetised by intramuscular injection of 66 mg·kg^−1^ ketamine and 33 mg·kg^−1^ xylazine. Using an operating microscope, 5 μL of a suspension that contained 1–10 × 10^12^ virions · μL^−1^ was intravitreally injected through the ora serrata by using a 10-μL Hamilton syringe with a 32-gauge needle (Hamilton Company, Reno, NV).

### Retrograde labelling of RGCs with the fluorescent tracer

For RGC retrograde labelling, we used one-year-old Thy1.2 TG (TG-ChR2) rats subjected to photoreceptor degeneration into which the mVChR1-Cherry gene had been transduced instead of the mVChR1-Venus gene in the same manner as described above. To examine the transduction efficiencies of *mVChR1* genes into RGCs, RGCs were retrograde-labelled with a fluorescent tracer by slowly injecting 4 μL of 2% aqueous fluorogold (Fluoro-Gold; Fluorochrome, Englewood, CO) containing 1% dimethyl sulfoxide into the superior colliculus using a Hamilton syringe with a 32-gauge needle.

### Measurement of transduction efficiencies

The right eyes were removed and fixed with 4% paraformaldehyde in 0.1 M phosphate buffer. Retinas were flat-mounted on slides, then covered with a mounting medium (Vectashild; Vector Laboratories, Burlingame, CA). For each slide, 3 areas in each quadrant (0.0988 mm^2^/area, 12 areas in total) were photographed using a fluorescence microscope (BZ-9000; KEYENCE, Osaka, Japan). Each image was obtained using Z-stacking and was stored. Cell counts were performed by image analysis (BZ-9000 software; KEYENCE, Osaka, Japan). Both the number of fluorogold-positive and fluorogold plus Cherry fluorescence double-positive RGCs were used to estimate the transduction efficiency of *mVChR1*. Furthermore, both the number of fluorogold-positive and fluorogold, Cherry, and Venus fluorescence triple-positive RGCs were used to estimate the percentage of RGCs double-positive for Cherry and Venus fluorescence.

### Cell culture

HEK293 cells were cultured in Minimum Essential Medium (Life Technologies Japan, Tokyo) supplemented with 10% foetal bovine serum under a 5% CO_2_ atmosphere at 37 °C. The culture medium was changed every 3 days and cells were passaged using a 0.02% ethylaminediaminetetraacetic acid/PBS solution.

### Establishment of ChR2 and mVChR1 expressing cells

Stably *ChR2* expressing cells were established by the method previously described^18^. In brief, the expression plasmid (pChR2-IRES-puro) was linearised using a restriction enzyme and electroporated into cultured HEK293 cells using the CUY21Pro-vitro system (Nepa Gene, Chiba, Japan). Transformants (HEK − ChR2) were selected in culture medium containing puromycin (0.1–30 μg/mL) for at least 10 d. Following the establishment of HEK-ChR2 cells, the linearised pAAV-mVChR1V vector was electroporated into stable transformant HEK-ChR2 cells. Venus-positive cells were sorted using a cell sorter (SH800; SONY, Tokyo, Japan) as cells stably expressing the *ChR2* and *mVChR1* genes (HEK − ChR2 + mVChR1).

### Patch clamp recordings

Photocurrents were recorded using an EPC-10 amplifier (HEKA Electronic, Lambrecht, Germany) under whole-cell patch clamping of isolated cells. Series resistance was compensated up to 70% to reduce series resistance errors. The data were collected by filtering at 10 kHz and sampled at 20 kHz. The internal solution contained 120 mM CsOH, 100 mM glutamate, 50 mM HEPES, 2.5 mM MgCl_2_, 2.5 mM MgATP, 5 mM Na_2_EGTA, and 1.2 mM leupeptin, with the pH adjusted to 7.2 for whole-cell current recordings. Tyrode’s solution contained 134 mM NaCl, 3 mM KCl, 2.5 mM CaCl_2_, 1.25 mM MgCl_2_, 4 mM NaOH, 10 mM HEPES, and 2 g/L glucose, with the pH adjusted to 7.4 by HCl. Photostimulation was performed on an inverted microscope (Eclipse; Nikon, Japan) equipped with a xenon lamp and electromagnetic shutter (Unibilitz, Rochester, NY). Various wavelengths (400, 450, 500, 550, and 600 nm) of light were produced by setting a band-pass filter (Fujifilm, Japan) in the carousel of the inverted microscope. The intensity of light at each wavelength was also adjusted to 1 μW/mm^2^ by setting an appropriate density filter for each wavelength into the carousel. The photocurrent was measured by 2–3 repetitions of a protocol, in which the wavelength was changed from 400 nm to 600 nm and the reverse order was applied with 1 s of light exposure every 20 s.

### Statistical analysis

Statistical analysis was performed using GraphPad Prism 4 (GraphPad Software, San Diego, CA). Data are expressed as the means ± SD. The statistical methods used were Tukey’s multiple comparison test or Dunnett’s multiple comparison test for the recording of VEPs and cell count data or relative analysis of VEPs, respectively.

## Additional Information

**How to cite this article**: Sato, M. *et al*. Visual Responses of Photoreceptor-Degenerated Rats Expressing Two Different Types of Channelrhodopsin Genes. *Sci. Rep.* **7**, 41210; doi: 10.1038/srep41210 (2017).

**Publisher's note:** Springer Nature remains neutral with regard to jurisdictional claims in published maps and institutional affiliations.
